# Effect of Admission Diabetes History on Safety and Efficacy of Early Tirofiban Infusion after Intravenous Thrombolysis in Ischaemic Stroke: a post-hoc analysis of the ASSET-IT trial

**DOI:** 10.1093/esj/aakag076

**Published:** 2026-07-13

**Authors:** Li Wang, Chunrong Tao, Cong Luo, Yuerong Chen, Yuyou Zhu, Pengfei Xu, Rui Li, Tianlong Liu, Chao Zhang, Jianlong Song, Jiangping Cui, Jun Sun, Wei Hu, Xinfeng Liu

**Affiliations:** Cheeloo College of Medicine, Shandong University, Jinan 250000, China; Department of Neurology, First Affiliated Hospital of the University of Science and Technology of China, Division of Life Sciences and Medicine, University of Science and Technology of China, Hefei 230001, China; Department of Neurology, First Affiliated Hospital of the University of Science and Technology of China, Division of Life Sciences and Medicine, University of Science and Technology of China, Hefei 230001, China; Department of Neurology, First Affiliated Hospital of the University of Science and Technology of China, Division of Life Sciences and Medicine, University of Science and Technology of China, Hefei 230001, China; The First Clinical Medical College of Anhui Medical University, Anhui Medical University, Hefei 230001, China; Department of Neurology, First Affiliated Hospital of the University of Science and Technology of China, Division of Life Sciences and Medicine, University of Science and Technology of China, Hefei 230001, China; Department of Neurology, First Affiliated Hospital of the University of Science and Technology of China, Division of Life Sciences and Medicine, University of Science and Technology of China, Hefei 230001, China; Department of Neurology, First Affiliated Hospital of the University of Science and Technology of China, Division of Life Sciences and Medicine, University of Science and Technology of China, Hefei 230001, China; Department of Neurology, First Affiliated Hospital of the University of Science and Technology of China, Division of Life Sciences and Medicine, University of Science and Technology of China, Hefei 230001, China; Department of Neurology, First Affiliated Hospital of the University of Science and Technology of China, Division of Life Sciences and Medicine, University of Science and Technology of China, Hefei 230001, China; Department of Neurology, First Affiliated Hospital of the University of Science and Technology of China, Division of Life Sciences and Medicine, University of Science and Technology of China, Hefei 230001, China; Department of Neurology, First Affiliated Hospital of the University of Science and Technology of China, Division of Life Sciences and Medicine, University of Science and Technology of China, Hefei 230001, China; Department of Neurology, First Affiliated Hospital of the University of Science and Technology of China, Division of Life Sciences and Medicine, University of Science and Technology of China, Hefei 230001, China; Department of Neurology, First Affiliated Hospital of the University of Science and Technology of China, Division of Life Sciences and Medicine, University of Science and Technology of China, Hefei 230001, China; Cheeloo College of Medicine, Shandong University, Jinan 250000, China; Department of Neurology, First Affiliated Hospital of the University of Science and Technology of China, Division of Life Sciences and Medicine, University of Science and Technology of China, Hefei 230001, China

**Keywords:** diabetes mellitus, stroke, thrombolysis, tirofiban

## Abstract

**Introduction:**

This study aimed to investigate how diabetes mellitus (DM) influences the efficacy of early tirofiban administration after intravenous thrombolysis in patients with acute ischaemic noncardioembolic stroke.

**Patients and methods:**

This was a post-hoc analysis of the ASSET-IT (Advancing Stroke Safety and Efficacy through Early Tirofiban Administration after Intravenous Thrombolysis) trial. Patients were categorised into DM and non-DM groups based on baseline diabetes history. The primary efficacy outcome was an excellent functional outcome at 90 days, defined as an mRS score of 0–1. Safety outcomes included sICH within 36 h, any ICH and all-cause mortality within 90 days. The effect of diabetes on treatment outcomes was assessed using multivariable regression models adjusting for relevant confounders.

**Results:**

A total of 832 patients at 38 centres were randomised in the ASSET-IT trial (414 to tirofiban, 418 to placebo). The median age was 69 years (IQR, 59–76); 301 (36.2%) were women. Overall, 191 patients (23.0%) had diabetes mellitus. Among patients without diabetes, an excellent functional outcome at 90 days occurred in 69.0% of patients in the tirofiban group and 55.8% in the placebo group, whereas among patients with diabetes, the corresponding rates were 55.0% and 52.0%, respectively. The nominal *P* value for the treatment-by-diabetes interaction was .009. The 90-day mortality was higher in the DM group receiving tirofiban (6.6% vs 2.0%; RR, 3.79; 95% CI, 0.84–17.05; *P* = .082) but not in the non-DM group (3.4% vs 4.4%; RR, 0.82; 95% CI, 0.40–1.70; *P* = .597).

**Conclusion:**

Exploratory post-hoc findings from the ASSET-IT trial suggest that the association between early tirofiban administration after intravenous thrombolysis and 90-day functional outcomes may differ according to diabetes status. These hypothesis-generating observations require confirmation in prospective studies with pre-specified subgroup analyses.

## Graphical Abstract

**Graphical Abstract:**
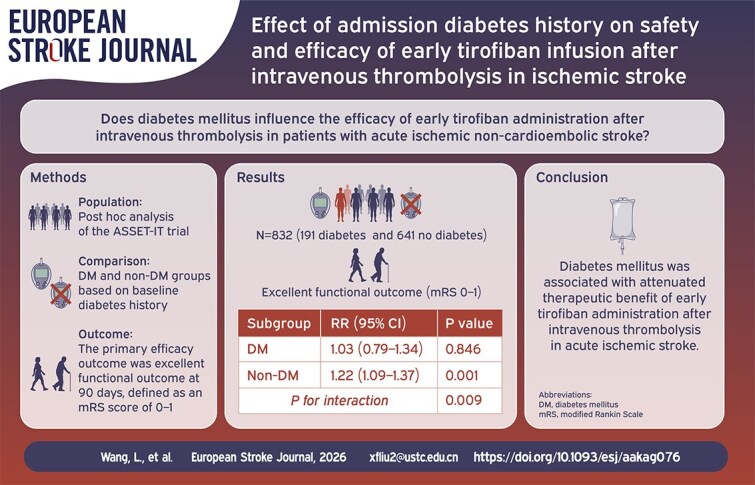

## Introduction

Acute ischaemic stroke (AIS) remains a leading cause of long-term disability and mortality globally.^1^ Intravenous thrombolysis and antiplatelet therapy are cornerstone interventions to restore cerebral blood flow and improve outcomes. Diabetes mellitus (DM) is associated with unfavourable outcomes in acute ischaemic stroke patients treated with intravenous thrombolysis.^2^^,^^3^ Some studies have shown that hyperglycaemia and diabetes mellitus lead to an increased risk of symptomatic intracerebral haemorrhage, poor functional outcomes and reduced recanalisation after treatment with intravenous thrombolysis therapy.^4–6^ Various mechanisms by which diabetes accelerates ischaemic tissue damage have been described, including impaired reactivity of the cerebral microvasculature, alterations in blood–brain barrier permeability, cortical acidosis, production of reactive oxygen and nitrogen species and hypercoagulability, which may ultimately increase infarct size and predispose patients to brain swelling and haemorrhagic transformation.^7–10^

Tirofiban, a potent glycoprotein receptor antagonist that can reduce macrovascular reocclusion, prevent microvascular thrombosis and improve cerebral blood flow in experimental models,^11^ has emerged as a valuable adjunctive therapy for AIS, particularly in patients ineligible for endovascular thrombectomy or those with progressive ischaemia.^12^ Although recent studies suggest that abnormal glucose metabolism may affect the efficacy of antiplatelet treatment,^13–15^ no study has yet examined whether DM modifies the treatment effect of tirofiban after intravenous thrombolysis. Therefore, we aimed to assess whether a history of diabetes at admission modifies the effect of tirofiban in AIS patients treated with intravenous thrombolysis through a post-hoc Analysis of the ASSET-IT (Advancing Stroke Safety and Efficacy through Early Tirofiban Administration after Intravenous Thrombolysis) trial^12^(ClinicalTrials.gov number: NCT06134622).

## Patients and methods

### Study population

The ASSET-IT trial was a multicentre, randomised, double-blind, placebo-controlled study that evaluated early intravenous tirofiban administration following intravenous thrombolysis in patients with acute ischaemic stroke who were not eligible for endovascular thrombectomy. The design and primary results of ASSET-IT have been reported previously.

For the present post-hoc analysis, we included all patients enrolled in the ASSET-IT trial who had complete baseline and outcome data. Patients were stratified according to the presence or absence of a history of diabetes mellitus, as recorded at baseline.

### Standard protocol approvals, registrations and patient consents

The study protocol was approved by the ethics committees or institutional review boards at all participating centres, and written informed consent was obtained from all patients or their legally authorised representatives before randomisation. The trial was registered at ClinicalTrials.gov (Identifier: NCT06134622).

### Baseline characteristics

Baseline demographic and clinical characteristics were collected at enrollment, including age, sex, body mass index (BMI), vascular risk factors (hypertension, hyperlipidaemia, coronary heart disease, smoking and previous ischaemic stroke or TIA), baseline blood glucose, pre-stroke mRS score, baseline NIHSS score, Alberta Stroke Program Early CT Score (ASPECTS), stroke aetiology and the type of intravenous thrombolytic agent used (alteplase or tenecteplase). Key time metrics—including onset-to-needle time, thrombolysis-to-randomisation time and thrombolysis-to-study-drug time—were recorded for all patients.

### Outcomes

The primary efficacy outcome was an excellent functional outcome at 90 days, defined as an mRS score of 0–1.

Secondary efficacy outcomes included good functional outcome (mRS 0–2), mRS score 0–3, Barthel Index at 90 days and health-related quality of life assessed by the EuroQol 5-Dimension questionnaire (EQ-5D).

Safety outcomes included sICH within 36 h, any ICH and all-cause mortality within 90 days.

### Statistical analysis

Patients were categorised into DM and non-DM groups. Baseline characteristics were summarised as medians (IQRs) for continuous variables and as numbers (percentages) for categorical variables. Between-group comparisons were performed using the χ^2^ test or Fisher’s exact test for categorical variables and the independent-sample *t* test or Mann–Whitney *U* test for continuous variables.

Effect modification by diabetes status was assessed by including a treatment-by-diabetes interaction term in the regression models. For binary outcomes, multivariable log-binomial regression models were used to estimate risk ratios and 95% CIs. The interaction models included treatment assignment, diabetes status, the treatment-by-diabetes interaction term, age, sex, baseline NIHSS score, pre-morbid mRS score and onset-to-needle time. The interaction coefficient was estimated on the log scale; exponentiating this coefficient represents the ratio of risk ratios for the treatment effect in patients with diabetes compared with those without diabetes. For continuous outcomes, multivariable linear regression models with the same covariates and interaction term were used.

Additional sensitivity analyses were performed to evaluate the potential influence of admission glucose. First, baseline glucose was added as a covariate to the adjusted treatment-by-diabetes interaction model for the primary outcome. Second, admission glucose was assessed as a potential effect modifier by including treatment-by-glucose interaction terms, with glucose modelled both as a continuous variable and as a categorical variable using a cut-off of 7.8 mmol/L to define acute hyperglycaemia.

All statistical analyses were conducted using Stata version 17.0 (StataCorp, College Station, TX, USA), and a 2-sided *P* value < .05 was considered statistically significant.

## Results

### Participants and characteristics

**Figure 1:**
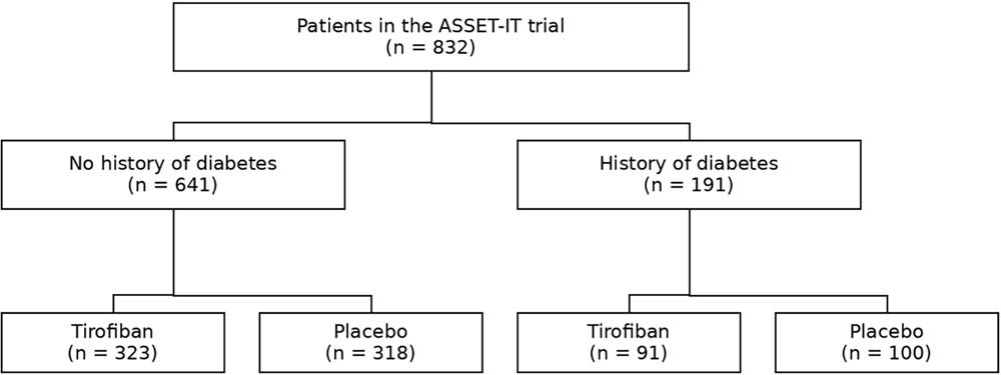
Flow diagram of the study population. Abbreviations: ASSET-IT = Advancing Stroke Safety and Efficacy through Early Tirofiban Administration after Intravenous Thrombolysis; DM = diabetes mellitus.

A total of 832 patients in the ASSET-IT trial were included in this post-hoc analysis, among whom 641 (77.0%) had no history of diabetes and 191 (23.0%) had diabetes (Figure 1). The median age was 69 years (IQR, 59–76), and 301 (36.2%) were women. Detailed baseline characteristics for patients with and without diabetes as well as across treatment groups are shown in Table 1.

**Table 1:** Baseline characteristics of patients in the 2 treatment groups according to the history of DM.

Variables	DM			Non-DM		
	Tirofiban	Placebo	*P* value	Tirofiban	Placebo	*P* value
**No. of patients**	91	100		323	318	
**Age, median (IQR), years**	67 (58, 74)	66 (58, 73)	.80	69 (59, 76)	70 (59, 77)	.59
**Male sex, no. (%)**	48 (52.8)	62 (62)	.20	212 (65.6)	209 (65.7)	.98
**mRS score of 1 before stroke**	4 (4.4)	5 (5)	.84	24 (7.4)	11 (3.5)	.03
**Medical history, no. (%)**						
** Hypertension**	84 (92.3)	84 (84)	.08	258 (79.9)	252 (79.3)	.84
** Coronary heart disease**	14 (15.4)	11 (11)	.37	35 (10.8)	30 (9.4)	.56
** Hyperlipidaemia**	20 (22)	20 (20)	.74	31 (9.6)	38 (12)	.34
** Previous ischaemic stroke or TIA**	28 (30.8)	37 (37)	.36	77 (23.8)	88 (27.7)	.27
**Smoking, no. (%)**	14 (15.4)	24 (24)	.28	68 (21.1)	74 (23.3)	.49
**Baseline glucose, mean (IQR), mmol/L**	9.8 (6.5, 12.8)	9.7 (7.3, 13.3)	.43	5.9 (5.0, 7.1)	5.6 (4.9, 6.6)	.04
**Cause of stroke, no. (%)**			.97			.72
** Large artery atherosclerosis**	53 (58.9)	57 (57.6)		194 (60.1)	180 (57.1)	
** Small artery occlusion**	34 (37.8)	39 (39.4)		105 (32.5)	112 (35.6)	
** Undetermined**	3 (3.3)	3 (3)		24 (7.4)	23 (7.3)	
**Baseline NIHSS, median (IQR)**	5 (5, 7)	6 (5, 9)	.10	6 (5, 9)	6 (5, 9)	.79
**Intravenous thrombolysis, no. (%)**			.47			.23
** Tenecteplase**	26 (28.6)	24 (24)		74 (22.9)	86 (27)	
** Alteplase**	65 (71.4)	76 (76)		249 (77.1)	232 (73)	
**Median ASPECTS (IQR)**	10 (9, 10)	10 (9.5, 10)	.24	10 (9, 10)	10 (9, 10)	.78
**BMI, median (IQR)**	23.9 (21.5, 26.0)	24.1 (22.0, 26.8)	.38	22.9 (20.2, 25.4)	22.9 (20.8, 25.7)	.45
**From stroke onset to thrombolysis, median (IQR), minutes**	179 (125, 222)	180 (130, 225)	.63	150 (110, 200)	167 (129, 220)	.004
**From thrombolysis completion to randomisation, median (IQR), minutes**	32 (12, 47)	38 (13, 49)	.54	29 (12, 47)	28 (12, 47)	.62
**From thrombolysis completion to administration of tirofiban or placebo, median (IQR), minutes**	44 (30, 55)	47 (30, 55)	.60	44 (25, 55)	42 (25, 54)	.49

Abbreviations: ASPECTS, Alberta Stroke Program Early Computed Tomography Score; BMI, body mass index; DM, diabetes mellitus.

Patients with diabetes had a higher body mass index (BMI, median 24.0 vs 22.9), and markedly higher prevalence of vascular risk factors, including hypertension (88% vs 79.6%), hyperlipidaemia (21% vs 10.7%), coronary heart disease (13.1% vs 9.9%) and previous ischaemic stroke or TIA (33.5% vs 25.7%), compared with those without diabetes. Median admission glucose was also substantially higher among patients with diabetes (≈9.8–9.7 mmol/L across groups) vs those without diabetes (≈5.6–5.9 mmol/L; *P* = .04). In contrast, the proportion of current smokers was lower in patients with diabetes than in those without diabetes (19.9% vs 22.4%). Patients with diabetes also had longer onset-to-needle times (median 179–180 min vs 150–167 min; *P* = .004), whereas times from thrombolysis completion to randomisation and to study drug administration were broadly similar across diabetes status (all *P* > .5).

By contrast, several baseline measures were balanced between the diabetes and non-diabetes groups: median NIHSS scores were 5–6 in patients with diabetes vs 6 in those without non-diabetes (all *P* > .1), and the distribution of stroke aetiologies was similar (large artery atherosclerosis was 58%–59% vs 57%–60%; small artery occlusion was 38%–39% vs 33%–36%). Alteplase was the major intravenous thrombolytic agent in both the diabetes and non-diabetes cohorts (71%–76% vs 73%–77%), with the remainder receiving tenecteplase.

In sensitivity analyses additionally adjusting for admission glucose, the treatment-by-diabetes interaction for the primary outcome was similar. When admission glucose was evaluated as a potential effect modifier, no statistically significant interaction was observed between treatment assignment and glucose level, either when glucose was modelled continuously or when acute hyperglycaemia was defined as glucose ≥ 7.8 mmol/L.

### Efficacy outcomes

**Figure 2:**
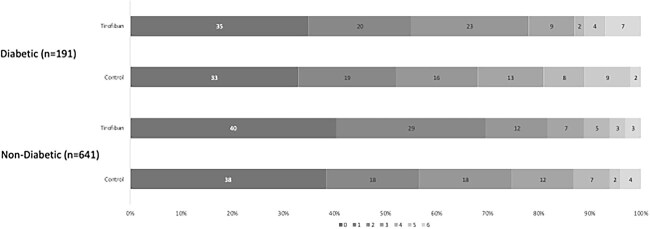
Distribution of mRS scores at 90 days stratified by diabetic history and tirofiban. Shown are scores on the mRS for the diabetic and non-diabetic patients in the tirofiban group and the placebo group. Scores range from 0 to 6, with 0 indicating no symptoms, 1 no clinically significant disability, 2 slight disability (patients are able to look after their own affairs without assistance but are unable to carry out all previous activities), 3 moderate disability (patients require some help but are able to walk unassisted), 4 moderately severe disability (patients are unable to attend to bodily needs without assistance and are unable to walk unassisted), 5 severe disability (patients require constant nursing care and attention) and 6 death.

Among patients without diabetes, 69.0% in the tirofiban group achieved mRS 0–1 at 90 days compared with 55.8% in the placebo group (RR, 1.22; 95% CI, 1.09–1.37; *P* = .001). In contrast, among patients with diabetes, the proportions were 55.0% vs 52.0% (RR, 1.03; 95% CI, 0.79–1.34; *P* = .846), showing no efficacy signal (Table 2). Similarly, in patients without diabetes, good functional outcome (mRS 0–2) at 90 days occurred in 81.1% vs 74.1%, respectively (RR, 1.09; 95% CI, 1.01–1.18; *P* = .028), whereas among those with diabetes the rates were 78.0% vs 68.0% (RR, 1.11; 95% CI, 0.95–1.31; *P* = .199). Compared with DM patients, more excellent functional outcome (mRS 0–1) at 90 days was achieved in non-DM patients treated with tirofiban administration after intravenous thrombolysis (interaction *P* = .009)(Figure 2). No significant between-treatment differences were observed for mRS 0–3 or for EQ-5D, regardless of diabetes status (Table 2). Findings were consistent in univariable analyses (Table S1). EQ-5D utility scores showed a ceiling effect, with median values close to or equal to 1.0 across all diabetes and treatment subgroups. Mean EQ-5D utility scores were additionally summarised to better characterise distributional differences. Among patients without diabetes, the mean EQ-5D utility score was 0.861 ± 0.274 in the tirofiban group and 0.832 ± 0.297 in the placebo group. Among patients with diabetes, the corresponding values were 0.841 ± 0.305 and 0.805 ± 0.304, respectively.

**Table 2:** The efficacy and safety outcomes of tirofiban vs placebo based on DM and non-DM stratification.

	DM				Non-DM				*P* for interaction
	Tirofiban	Placebo	Effect size (95% CI)	*P* value	Tirofiban	Placebo	Effect size (95% CI)	*P* value	
**Primary outcome no. (%)**									
**mRS 0–1**	50 (55)	52 (52)	1.03 (0.79–1.34)	.846	223 (69.0)	177 (55.8)	1.22 (1.09–1.37)	.001	.009
**Secondary outcomes no. (%)**									
**mRS 0–2**	71 (78.0)	68 (68.0)	1.11 (0.95–1.31)	.199	262 (81.1)	235 (74.1)	1.09 (1.01–1.18)	.028	.159
**mRS 0–3**	79 (86.8)	81 (81.0)	1.05 (0.94–1.18)	.397	286 (88.5)	273 (86.1)	1.03 (0.97–1.08)	.351	.251
**Barthel**	66 (72.5)	67 (67.0)	1.06 (0.88–1.27)	.563	253 (78.3)	227 (71.6)	1.09 (1.00–1.18)	.04	.069
**EQ-5D**	1.0 (0.9–1.0)	1.0 (0.7–1.0)	0.02 (−0.06–0.11)	.598	1.0 (0.9–1.0)	1.0 (0.8–1.0)	0.03 (−0.01–0.07)	.207	.129
**NIHSS score**									
**Median score at 24–72 h**	4.0 (2.0–6.0)	4.0 (2.0–6.0)	0.11 (−1.30–1.52)	.88	3.0 (1.0–6.0)	3.0 (1.0–6.0)	0.1 (−0.56–0.75)	.773	.054
**Median score at 5–7 days or discharge**	2.0 (1.0–4.0)	3.0 (1.0–6.0)	−0.3 (−1.36–0.77)	.588	2.0 (1.0–4.0)	2.0 (1.0–4.0)	0.04 (−0.60–0.67)	.912	.170
**Safety outcomes**									
**death**	6 (6.6)	2 (2.0)	3.79 (0.84–17.05)	.082	11 (3.4)	14 (4.4)	0.82 (0.40–1.70)	.597	.023
**ICH**	7 (7.7)	1 (1.0)	5.27 (0.66–42.04)	.117	15 (4.6)	13 (4.1)	1.1 (0.53–2.29)	.79	.092
**Symptomatic within 36 h**	2 (2.2)	0 (0.0)	NA	NA	5 (1.6)	0 (0.0)	NA	NA	NA
**Asymptomatic**	5 (5.5)	1 (1.0)	3.42 (0.39–29.77)	.266	10 (3.1)	13 (4.2)	0.77 (0.33–1.77)	.536	.128

Abbreviations: DM, diabetes mellitus; EQ-5D, EuroQol 5-Dimension questionnaire.

Among patients without diabetes, 15.5% had admission hyperglycaemia, defined as an admission glucose level ≥ 7.8 mmol/L. In sensitivity analyses additionally adjusting for admission glucose, the treatment effect of tirofiban for an excellent functional outcome at 90 days remained similar (adjusted RR, 1.24; 95% CI, 1.10–1.40; *P* < .001). When admission glucose was evaluated as a potential effect modifier, no statistically significant treatment-by-glucose interaction was observed when glucose was modelled as a continuous variable (*P* for interaction = .067) (Table S2).

### Safety

In patients with diabetes, 90-day mortality was numerically higher with tirofiban (6.6%) compared with placebo (2.0%) (RR, 3.79; 95% CI, 0.84–17.05; *P* = .082), whereas in those without diabetes the corresponding rates were 3.4% vs 4.4% (RR, 0.82; 95% CI, 0.40–1.70; *P* = .597), with a significant interaction between diabetes status and treatment effect (*P* for interaction = .023) (Table 2). Intracranial haemorrhage occurred in 7.7% vs 1.0% among diabetes patients and 4.6% vs 4.1% among non-diabetes patients, respectively (*P* for interaction = .092). Symptomatic ICH within 36 h was uncommon in both groups (2.2% vs 0% among diabetes patients and 1.6% vs 0% among non-diabetes patients), and estimates tended to be imprecise due to low event numbers.

## Discussion

In this post-hoc analysis of the ASSET-IT trial, approximately one-quarter of patients had a history of diabetes mellitus at admission. We observed that the functional benefit associated with early tirofiban administration following intravenous thrombolysis appeared to differ according to diabetes status. Specifically, patients without diabetes experienced higher rates of disability-free and functionally independent outcomes at 90 days with tirofiban compared with placebo, whereas no such benefit was observed among patients with diabetes. These findings suggest that diabetes may act as a potential effect modifier of adjunctive antiplatelet therapy following thrombolysis.

Admission hyperglycaemia was also observed in a proportion of patients without known diabetes and may reflect acute stress hyperglycaemia. Although sensitivity analyses additionally adjusting for admission glucose produced similar treatment-effect estimates, acute glycaemic status may still contribute to residual confounding and cannot fully substitute for measures of chronic glycaemic exposure, such as HbA1c or diabetes duration.

Although the proportion of large artery atherosclerosis was similar between patients with and without diabetes, this aetiological category may not fully capture differences in vascular biology. Diabetes may be associated with more diffuse atherosclerosis, a greater intracranial atherosclerotic burden, more severe stenosis, endothelial dysfunction or more vulnerable plaques. These factors could influence thrombus formation, reperfusion stability and the response to adjunctive antiplatelet therapy. However, detailed data on stenosis severity, plaque morphology and intracranial vs extracranial atherosclerotic distribution were not systematically collected, and these mechanisms could not be directly tested in the present analysis.

Several diabetes-related mechanisms may partly explain the attenuated apparent treatment benefit observed among patients with diabetes. Diabetes is associated with endothelial dysfunction, impaired microvascular reactivity, increased platelet activation and accelerated platelet turnover.^16^ Experimental and translational studies suggest that diabetes-related inflammatory signalling may promote reticulated thrombocytosis and an increased proportion of immature platelets.^17^ Reticulated platelets are more reactive and may enter the circulation after antiplatelet therapy has been initiated, which could contribute to altered responsiveness to antiplatelet agents, including glycoprotein IIb/IIIa inhibition. In addition, hyperglycaemia and diabetes are associated with increased plasminogen activator inhibitor-1 activity, glycation-related alterations in fibrin structure and impaired fibrinolysis, potentially promoting thrombus persistence or early reformation after intravenous thrombolysis.^18–23^

These mechanisms may provide biological plausibility for the observed exploratory subgroup pattern; however, platelet turnover, fibrinolytic markers and thrombus characteristics were not measured in this study.

With respect to safety, the numerically higher mortality observed among patients with diabetes receiving tirofiban should be interpreted cautiously because it was based on a small number of events and wide confidence intervals. Although diabetes-related endothelial dysfunction, impaired fibrinolysis, renal dysfunction and other comorbidities may contribute to worse outcomes and bleeding vulnerability, our data cannot establish a causal mechanism linking tirofiban to mortality in this subgroup. Larger studies with adequate power are required to clarify the safety profile of tirofiban in patients with diabetes.^24^^,^^25^

Although multivariable models adjusted for key prognostic variables, including onset-to-needle time and pre-morbid mRS scores, residual imbalance and unmeasured confounding cannot be excluded. Patients with diabetes had longer onset-to-needle times, which may reflect atypical symptom recognition, greater comorbidity burden or prehospital delay and may have influenced functional outcomes.

Our findings align with prior evidence demonstrating that diabetes is associated with poorer outcomes after intravenous thrombolysis. However, few studies have specifically examined whether diabetes modifies the response to adjunctive antiplatelet therapy in the early post-thrombolysis period. Because this was a post-hoc subgroup analysis that was not pre-specified in the ASSET-IT protocol, randomisation may not be fully preserved within diabetes subgroups and residual confounding cannot be excluded. In addition, multiple outcomes and subgroup comparisons were assessed without formal adjustment for multiplicity; therefore, the reported *P* values should be considered nominal, and the possibility of type I error should be acknowledged. These findings should be interpreted as exploratory and hypothesis-generating rather than confirmatory.

Several limitations should be acknowledged. First, this was a post-hoc subgroup analysis that was not pre-specified in the ASSET-IT protocol. Therefore, randomisation may not be fully preserved within diabetes subgroups, residual confounding cannot be excluded and causality cannot be established. Multiple outcomes and subgroup comparisons were assessed without formal adjustment for multiplicity; thus, the reported *P* values should be considered nominal, and the findings should be interpreted as exploratory and hypothesis-generating. Second, the diabetes subgroup was relatively small, including only 191 patients, and the analysis was underpowered, particularly for safety outcomes such as mortality and ICH. Baseline differences, including longer onset-to-needle times among patients with diabetes and an imbalance in admission glucose among patients without diabetes, may have influenced outcomes despite covariate adjustment. Although sensitivity analyses additionally adjusting for admission glucose produced similar findings, residual confounding related to acute glycaemic status remains possible. Third, diabetes was defined according to baseline medical history and analysed as a binary variable. Detailed data on diabetes type, duration, HbA1c, antidiabetic treatment and diabetes-related microvascular or macrovascular complications were not systematically collected. Serum creatinine was available in most patients, but detailed kidney disease and bleeding-risk profiles were limited. In addition, vascular features such as stenosis severity, plaque vulnerability and intracranial vs extracranial atherosclerotic distribution were not systematically assessed. Furthermore, EQ-5D utility scores showed a pronounced ceiling effect, with median values close to or equal to 1.0 across groups, which may have limited the ability of this measure to detect between-group differences in health-related quality of life in this cohort. Therefore, EQ-5D results should be interpreted cautiously and were considered supportive rather than central to the present exploratory analysis. Finally, this study was conducted exclusively in Chinese centres, which may limit the generalisability of the findings to European and other non-Chinese populations. In our cohort, large artery atherosclerosis accounted for approximately 58%–60% of stroke aetiologies, a distribution that may differ from that observed in typical European stroke cohorts. Differences in genetic background, vascular risk-factor profiles, comorbidity patterns, stroke mechanisms, healthcare infrastructure, prehospital pathways and treatment timing may influence both baseline prognosis and response to early adjunctive antiplatelet therapy. Therefore, direct extrapolation of these exploratory findings to Western populations should be made cautiously, and validation in more diverse populations is warranted.

## Conclusion

This post-hoc analysis of the ASSET-IT trial suggests that the association between early tirofiban administration after intravenous thrombolysis and functional outcomes may differ according to diabetes status, with greater benefit observed among patients without diabetes. These findings indicate that diabetes could represent a potential effect modifier of treatment response. Prospective studies with pre-specified stratification by diabetes status are required to validate these observations and to guide individualised therapeutic strategies.

## Supplementary Material

Table_S1_aakag076

Table_S2_aakag076

## Data Availability

De-identified individual participant data that underlie the reported results are available from the corresponding author upon reasonable request and with appropriate institutional approvals.
