# Supplementary material for: Effect of Admission Diabetes History on Safety and Efficacy of Early Tirofiban Infusion after Intravenous Thrombolysis in Ischaemic Stroke: a post-hoc analysis of the ASSET-IT trial
Source: Eur Stroke J. 2026 Jul 13;11(7):aakag076. doi: 10.1093/esj/aakag076 (PMC13358872; doi:10.1093/esj/aakag076)
Supplement: Table_S1_aakag076 [file table_s1_aakag076.docx]

**Table S1.** Univariable analysis for supplementary analysis between DM and non-DM history patients

|  | DM | | Non-DM | |
| --- | --- | --- | --- | --- |
| P | **Effect size(95% CI)** | ***P* value** | **Effect size(95% CI)** | ***P* value** |
| Primary outcome |  |  |  |  |
| mRS 0-1 | 1.06 (0.81–1.38) | 0.683 | 1.24 (1.09–1.40) | 0.001 |
| Secondary outcomes |  |  |  |  |
| mRS 0-2 | 1.15 (0.96–1.36) | 0.120 | 1.09 (1.01–1.19) | 0.035 |
| mRS 0-3 | 1.07 (0.95–1.21) | 0.274 | 1.03 (0.97–1.09) | 0.357 |
| Barthel | 1.08 (0.90–1.30) | 0.406 | 1.09 (1.00–1.20) | 0.051 |
| EQ-5D | 0.04 (-0.05–0.12) | 0.419 | 0.03 (-0.01–0.07) | 0.189 |
| NIHSS score |  |  |  |  |
| Median score at 24-72h | -0.14 (-1.75–1.47) | 0.862 | 0.15 (-0.59–0.89) | 0.683 |
| Median score at 5-7 days or discharge | -0.81 (-1.97–0.34) | 0.168 | 0.1 (-0.57–0.76) | 0.777 |
| Safety outcomes |  |  |  |  |
| death | 3.3 (0.68–15.92) | 0.138 | 0.77 (0.36–1.68) | 0.516 |
| Intracranial hemorrhage | 7.69 (0.96–61.32) | 0.054 | 1.14 (0.55–2.35) | 0.731 |
| Asymptomatic hemorrhage | 5.27 (0.63–44.29) | 0.126 | 0.76 (0.34–1.70) | 0.501 |

**Abbreviations: DM，Diabetes Mellitus; mRS，modified Rankin Scale；EQ-5D，EuroQol-5D Questionnaire；NIHSS, National Institutes of Health Stroke Scale.**
