# Supplementary material for: Effect of Admission Diabetes History on Safety and Efficacy of Early Tirofiban Infusion after Intravenous Thrombolysis in Ischaemic Stroke: a post-hoc analysis of the ASSET-IT trial
Source: Eur Stroke J. 2026 Jul 13;11(7):aakag076. doi: 10.1093/esj/aakag076 (PMC13358872; doi:10.1093/esj/aakag076)
Supplement: Table_S2_aakag076 [file table_s2_aakag076.docx]

**Table S2. Additional adjustment for baseline glucose between DM and non-DM history patients.**

|  | *DM* | | *Non-DM* | |  |
| --- | --- | --- | --- | --- | --- |
|  | **Effect size(95% CI)** | ***P* value** | **Effect size(95% CI)** | ***P* value** | ***P for interaction*** |
| Primary outcome no. (%) |  |  |  |  |  |
| mRS 0-1 | 0.97 (0.74–1.26) | 0.794 | 1.24 (1.10–1.40) | <0.001 | 0.064 |
| Secondary outcomes no. (%) |  |  |  |  |  |
| mRS 0-2 | 1.11 (0.94–1.31) | 0.216 | 1.12 (1.03–1.21) | 0.007 | 0.751 |
| mRS 0-3 | 1.07 (0.95–1.19) | 0.273 | 1.05 (0.99–1.11) | 0.078 | 0.966 |
| Barthel | 1.05 (0.87–1.27) | 0.601 | 1.12 (1.02–1.22) | 0.012 | 0.405 |
| EQ-5D | 0.02 (-0.06–0.11) | 0.598 | 0.03 (-0.01–0.07) | 0.207 | 0.586 |
| Median score at 24-72h | 0.59 (-0.83–2.00) | 0.418 | -0.26 (-0.89–0.38) | 0.426 | 0.177 |
| Median score at 5-7 days or discharge | 0.24 (-0.66–1.15) | 0.598 | -0.15 (-0.76–0.47) | 0.644 | 0.616 |
| Safety outcomes |  |  |  |  |  |
| death | 2.99 (0.81–11.06) | 0.1 | 0.78 (0.34–1.78) | 0.557 | 0.138 |
| Intracranial hemorrhage | 4.78 (0.57–39.86) | 0.148 | 1.06 (0.47–2.36) | 0.889 | 0.074 |
| Symptomatic within 36 hr | NA | NA | NA | NA | NA |
| Asymptomatic | 3.05 (0.34–27.63) | 0.322 | 0.74 (0.30–1.84) | 0.52 | 0.082 |

**Abbreviations: DM，Diabetes Mellitus; mRS，modified Rankin Scale；EQ-5D，EuroQol-5D Questionnaire；NIHSS, National Institutes of Health Stroke Scale.**
